# Immersive Virtual Reality and Ocular Tracking for Brain Mapping During Awake Surgery: Prospective Evaluation Study

**DOI:** 10.2196/24373

**Published:** 2021-03-24

**Authors:** Morgane Casanova, Anne Clavreul, Gwénaëlle Soulard, Matthieu Delion, Ghislaine Aubin, Aram Ter Minassian, Renaud Seguier, Philippe Menei

**Affiliations:** 1 Équipe Facial Analysis Synthesis & Tracking Institue of Electronics and Digital Technologies CentraleSupélec Rennes France; 2 Département de Neurochirurgie Centre hospitalier universitaire d'Angers Angers France; 3 Centre de Recherche en Cancérologie et Immunologie Nantes Angers Université d'Angers Centre hospitalier universitaire d'Angers Angers France; 4 Département d'Anesthésie-Réanimation Centre hospitalier universitaire d'Angers Angers France

**Keywords:** virtual reality, eye tracking, brain mapping, awake surgery, visuospatial cognition, nonverbal language, mobile phone

## Abstract

**Background:**

Language mapping during awake brain surgery is currently a standard procedure. However, mapping is rarely performed for other cognitive functions that are important for social interaction, such as visuospatial cognition and nonverbal language, including facial expressions and eye gaze. The main reason for this omission is the lack of tasks that are fully compatible with the restrictive environment of an operating room and awake brain surgery procedures.

**Objective:**

This study aims to evaluate the feasibility and safety of a virtual reality headset equipped with an eye-tracking device that is able to promote an immersive visuospatial and social virtual reality (VR) experience for patients undergoing awake craniotomy.

**Methods:**

We recruited 15 patients with brain tumors near language and/or motor areas. Language mapping was performed with a naming task, DO 80, presented on a computer tablet and then in 2D and 3D via the VRH. Patients were also immersed in a visuospatial and social VR experience.

**Results:**

None of the patients experienced VR sickness, whereas 2 patients had an intraoperative focal seizure without consequence; there was no reason to attribute these seizures to virtual reality headset use. The patients were able to perform the VR tasks. Eye tracking was functional, enabling the medical team to analyze the patients’ attention and exploration of the visual field of the virtual reality headset directly.

**Conclusions:**

We found that it is possible and safe to immerse the patient in an interactive virtual environment during awake brain surgery, paving the way for new VR-based brain mapping procedures.

**Trial Registration:**

ClinicalTrials.gov NCT03010943; https://clinicaltrials.gov/ct2/show/NCT03010943.

## Introduction

Brain mapping by direct electrical stimulation (DES) during awake craniotomy is currently a standard procedure that reduces the risk of permanent neurological deficits and increases the extent of tumor resection and the success of epilepsy surgery [1]. This technique aims to temporarily inactivate a discrete brain area using DES while the patient performs a task. If the patient’s performance in the task decreases during inactivation, then the region of the brain explored is considered eloquent for the task and is preserved.

Verbal language, which is controlled by the dominant hemisphere, is widely mapped in this way [2]. Other cognitive functions, such as visuospatial cognition and nonverbal language, including facial expressions and eye gaze, which play an important role in social interaction, have been explored by only a few groups [3]. One of the main reasons for the lack of mapping these functions is the difficulty in adapting classic bedside neuropsychological tasks to awake surgery conditions. In particular, the patient must give an unambiguous answer within 5 seconds, which is the maximum duration of DES. Therefore, there is a need for new tools, allowing complex neuropsychological evaluations, that are compatible with the restrictive environment of an operating room and awake brain surgery procedures.

A few years ago, we began exploring the feasibility of testing cognitive functions during awake craniotomy by immersing the patient in virtual situations with a virtual reality headset (VRH). We have developed several different approaches using different types of headsets and software. The first virtual reality (VR) tasks were developed with the aim of preventing postoperative hemianopsia and unilateral neglect [4]. However, the risk of VR sickness or VRH-induced seizures raised concerns for all screen-based video games [5-8]. Therefore, we performed an initial study evaluating the tolerance of a wireless, low-cost, high-quality, and customizable device: the Samsung Gear VR combined with a Samsung S7 smartphone [9]. This trial, on 30 patients, showed that VRH use and immersive virtual experiences were both feasible and safe for patients undergoing awake craniotomy and brain mapping using DES. Various VR experiences were tested, including a picture-naming task, DO 80 [10] and, a social VR task, vTime, simulating virtual social interactions with an avatar piloted by a neuropsychologist, who also wore a VRH [11]. Toward the end of this safety study, a new VRH with higher performance, the HTC VIVE (HTC Corporation), including an eye-tracking device, was released. Therefore, we decided to prolong the study, including 15 more patients using this new device, to analyze the feasibility of using eye tracking during awake craniotomy. Furthermore, during the initial trial, we experienced some limitations to the use of the social VR task vTime, precisely because of the lack of control of all potent nonverbal language cues, including facial expressions and eye gaze. Therefore, we decided to pursue our efforts to explore visuospatial cognition and nonverbal language during awake surgery by developing an interactive VR task capable of analyzing these functions simultaneously. The possibilities and limitations of this new visuospatial and social VR experience are presented in this paper.

## Methods

### Study Design

We performed a single-center, prospective, and open-label study, and the study protocol was evaluated and approved by the Agence Nationale de Sécurité du Médicament et des produits de santé, the local ethics committee, and Commission Nationale de l'Informatique et des Libertés. All patients signed a written informed consent form before inclusion in the study. This study was registered at ClinicalTrials.gov (NCT03010943). As indicated above, an amendment was requested and accepted to assess the feasibility of using eye tracking with a VRH during awake craniotomy and to explore the possibilities and limitations of the visuospatial and social VR experience. During the extension of the study, we continued to use questionnaires completed by the patient and medical professionals to assess tolerance (discomfort, nausea, vomiting, and visual-vestibular-somatosensory conflict) and satisfaction (Multimedia Appendix 1). The occurrence of electroencephalogram (EEG) modifications (afterdischarge) or intraoperative seizures (IOSs) was also recorded.

The inclusion criteria were as follows: patients aged >18 years hospitalized for a brain tumor near language and/or motor areas (determined by neuropsychological evaluation and resting-state functional magnetic resonance imaging [fMRI]) in the left or right hemisphere who gave written informed consent. The exclusion criteria were all contraindications for awake surgery (cognitive impairment, whether related to the surgical lesion, aphasia, or morbid anxiety). A total of 15 patients were included in the extension study.

### Virtual Reality Headset

This study was performed with a Tobii Pro VR Integration, an eye-tracking retrofitted HTC VIVE wired to a computer connected to a neuronavigational system (Brainlab). The VRH has a visual field of 110°, an adjustable interpupillary distance, a latency <20 milliseconds, a refresh rate of 90 Hz, a resolution of 2160×1200 pixels, and adjustable focus. The VRH includes the eye-tracking systems developed by Tobii Pro for research purposes (Tobii Pro), which collects various types of eye movement data, such as gaze origin and direction, pupil position, and absolute pupil size with an accuracy of 0.5° visual angle at a rate of 120 Hz. What the patient sees in the VRH is visualized on one of the screens of the neuronavigational system.

### VR Tasks

**Figure 1:**
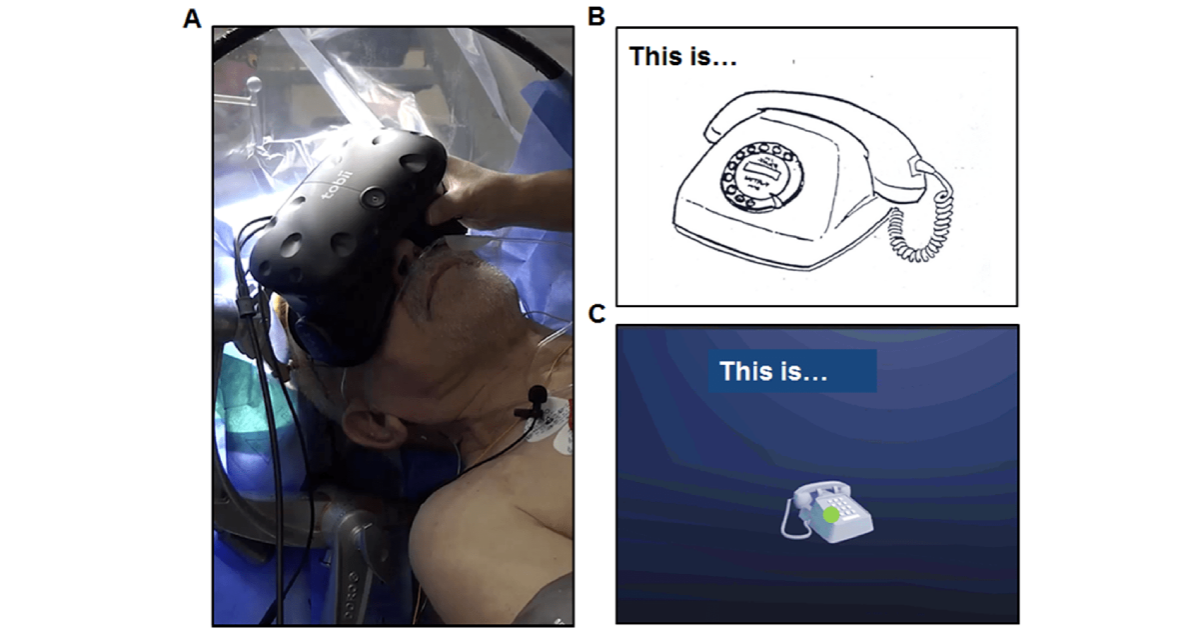
(A) Patient wearing the virtual reality headset. (B) and (C) Example of the item “phone” in the DO 80 naming task presented in 2D (B) and 3D (C) with the virtual reality headset. The green spot indicates the patient’s gaze.

The picture-naming task, DO 80, was implemented in the VRH in 2 versions [10]. The first version, in 2D (2D VR), included the same images as the classical naming task DO 80 presented with a computer tablet (an image with the sentence “this is...”). The second version included the same items but in 3D (3D VR), rotating in a virtual, empty space (Figure 1). The object-naming task is simple, making it possible to identify various types of errors, and is the most widely used task for language mapping [12,13].

**Figure 2:**
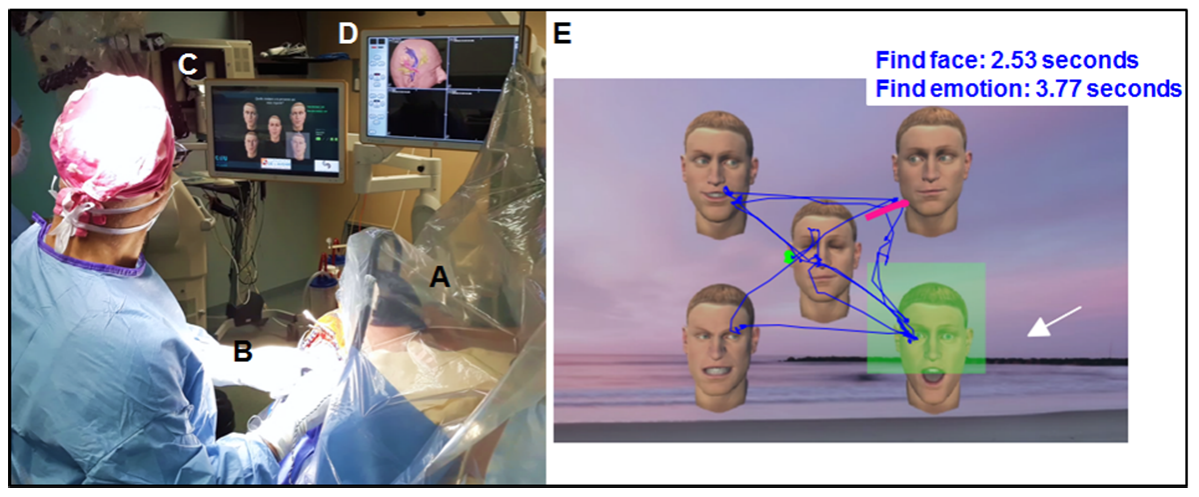
Left: view of the operating room during the procedure. (A) Head of the patient wearing the virtual reality headset; (B) application of direct electrical stimulation to the exposed brain; (C) screen showing what the patient sees in the virtual reality headset, his gaze materialized by a green spot; (D) neuronavigational system showing brain white matter fascicles and the position of the electrode. Right: example of a layout after the virtual reality task simulating a visuospatial and social experience. (E) The image that is visualized and analyzed on the screen (C). The movement of the patient’s gaze is visualized as a blue line (with the starting point in green and the endpoint in pink). The green box indicates the avatar making eye contact. The white arrow indicates the avatar on which the patient focuses for more than 0.6 seconds (triggering the expression of a dynamic facial emotion). In this example, the patient identified the avatar making eye contact in 2.53 seconds and indicated the emotion expressed 3.77 seconds later.

The new visuospatial and social VR experience that we have developed uses animated synthetic characters (avatars; Figure 2). The scene shows 5 avatars in front of a landscape background. The avatar in the center has his eyes closed and the others in the 4 quadrants of the visualized VRH field are looking in different directions. Patients were asked to search for the avatar staring at them. Less than a second (0.6 seconds) after visual contact is established, the avatar expresses a dynamic facial emotion that the patient is asked to identify and describe: joy, surprise, or anger. If the patient stares at the wrong avatar, a dynamic facial emotion is nevertheless initiated. Facial expressions were enacted by an actor and then transferred onto the avatars with a professional tool from the game or cinema industry [14]. For each test performed with this VR task, the medical team can follow the gaze of the patient directly, materialized as a green point on one of the screens of the neuronavigational system. This eye tracking reveals whether patients have difficulties exploring the space, have difficulties locating the face of the avatar looking at them, or fail to recognize the facial emotion. At the end of each test, the gaze layout, the time to perform the task, and the answer given by the patient were recorded (Figure 2).

### Operative Procedure

The procedure has been described in detail elsewhere [9]. Before surgery, all patients underwent a neuropsychological evaluation and fMRI and were trained in the use of VR tasks in the context of awake surgery. General anesthesia was administered via a laryngeal airway mask during surgery. Patients were positioned in a supine or lateral position, according to the location of the tumor, with a rigid pin fixation of the head. The scalp was infiltrated with a local anesthetic. Once the craniotomy, guided by neuronavigation, was completed and the dura had been opened, after infiltration, the patient was woken up. EEG signals were recorded with a four-plot subdural electrode (four-channel Eclipse neurovascular workstation, Medtronic XOMED, Inc). DES was performed with a bipolar electrode (tip-to-tip distance: 5 mm), delivering a biphasic current (parameters: 60 Hz, 1-millisecond single-pulse duration, and current amplitude from 1 mA-8 mA). Stimulation was applied to every 1 cm^2^ of the exposed cerebral cortex. If a functional cortical area was identified, a minimum margin of 1 cm was observed during the resection. We continued monitoring movement and/or spontaneous language during tumor resection, and a second mapping with subcortical electrostimulation was performed if necessary.

Brain mapping for language was performed with the picture-naming task, DO 80, using a computer tablet. Sites were identified as language sites if interference (speech arrest, anomia, dysarthria, semantic or phonemic paraphasia, or delayed naming >5 seconds) was detected in at least 3 meticulous tests (not necessarily consecutively) and were tagged on the cortex. A second round of mapping was then performed using the VRH, with the 2D DO 80 and then with the 3D DO 80 task. Differences in responses were carefully noted. Depending on the location of the tumor, other tests were proposed on a computer tablet (spontaneous speech production, counting, reading, etc).

The visuospatial and social VR experience with avatars was included in brain mapping by DES when considered necessary to test these functions. In other situations, this VR task was proposed for patients without DES, generally during the closure period. The 4 quadrants of the visual field and all emotions (joy, surprise, or anger) were presented randomly to the patient. As for language mapping, a site was identified as eloquent if interference through DES (difficulties exploring the space, difficulties locating the avatar making eye contact, or failure to recognize the facial emotion, resulting in a delay or lack of response from the patient) was detected 3 times. Once the task was completed, the gaze layout, the time to perform the task, and the patient’s answer were recorded (Figure 2).

The entire procedure was performed in the presence of an engineer and a neuropsychologist. Heart rate, blood pressure, and EEG signals were recorded continuously during the procedure. Spontaneous or stimulation-induced afterdischarges recorded on EEG, were defined as 2 consecutive spikes or sharp waves distinct from background activity. Any drug administration differing from that laid out in the predefined protocol was noted. Tolerance was also assessed with a questionnaire completed by the patient, the anesthetist, the neuropsychologist, and the neurosurgeon.

## Results

Baseline characteristics of the 15 patients are presented in Table 1. A total of 15 patients (8 men and 7 women) with a median age of 52 years (range 25-73 years) underwent the procedure; 2 of the patients were left-handed, and 13 were right-handed. The tumor was in the left hemisphere in 11 patients and the right hemisphere in 4 patients. Patients were initially hospitalized for seizures (9/15, 60%), aphasia (1/15, 7%), or alexia (1/15, 7%). The tumor was discovered through monitoring of the primary cancer in 27% (4/15) of the patients. The mean tumor diameter was 38.6 millimeters (range 25-50 millimeters). Tumors were located in the frontal lobe (8/15, 53%), parietal lobe (4/15, 27%), temporoparietal junction (2/15, 13%), or frontotemporal insular cortex (1/15, 7%). The lesions were grade 2 oligodendroglioma (1/15, 7%), anaplastic oligodendroglioma (2/15, 13%), anaplastic astrocytoma (6/15, 40%), glioblastoma (3/15, 20%), or metastasis (3/15, 20%).

**Table 1:** Baseline characteristics of the 15 patients and the virtual reality tasks they performed.

Patient	Sex	Age (years)	Handedness	Diagnosis	Hemisphere	Lobe	Preoperative training	Brain mapping
1	Male	68	Left	Metastasis	Left	Parietal	Task 1^a^ and task 2^b^	Task 1 and task 2
2	Male	41	Right	Oligodendroglioma II	Right	Frontal	Task 1 and task 2	Motor and task 2
3	Female	25	Right	Astrocytoma III	Left	Frontal	Task 1	Motor and task 1
4	Female	66	Right	Oligodendroglioma III	Right	Frontal	Task 2	Motor
5	Male	39	Left	Astrocytoma III	Right	Frontal	Task 1 and task 2	Motor and task 1 and task 2
6	Female	60	Right	Glioblastoma	Left	Temporoparietal	Task 1	Task 1
7	Male	48	Right	Oligodendroglioma III	Left	Frontal	Task 1 and task 2	Task 1 and task 2
8	Female	53	Right	Glioblastoma	Left	Parietal	Task 1	Task 1
9	Male	68	Right	Glioblastoma	Left	Frontal	Task 1 and task 2	Task 1
10	Male	73	Right	Metastasis	Left	Frontal	Task 1 and task 2	Task 1 and task 2
11	Female	47	Right	Astrocytoma III	Left	Parietal	Task 1 and task 2	Motor and task 1
12	Male	61	Right	Metastasis	Left	Temporoparietal	Task 1 and task 2	Task 1
13	Male	43	Right	Astrocytoma III	Left	Parietal	Task 1 and task 2	Task 1 and task 2
14	Female	53	Right	Astrocytoma III	Left	Frontotemporal insular	Task 1 and task 2	Task 1
15	Female	41	Right	Astrocytoma III	Right	Frontal	Task 2	Task 2

^a^Task 1: DO 80 (tablet, 2D virtual reality, and 3D virtual reality).

^b^Task 2: visuospatial and social virtual reality experience.

Only 3 patients had experienced VR before inclusion. Before surgery, 13 patients were trained with the DO 80 task (tablet, 2D VR, and 3D VR), and 12 patients were trained with the VR task simulating a visuospatial and social experience (Table 1). No preoperative difficulties were observed with the DO 80 task, but 2 patients (patients 4 and 12) experienced difficulties with visuospatial and social VR tasks. In particular, they encountered problems in finding the avatar looking at them. For these 2 patients, this task was not applied during awake surgery (Table 1).

The mean duration of surgery was 4 hours and 23 minutes (range 3 h and 6 min-5 h and 30 min), with a mean duration of the awake phase of 2 hours and 20 minutes (range 25 min-4 h). The mean intensity of DES was 1.9 mA (range 1-4 mA), and the mean total duration of VRH use per patient was 11 minutes in 2 to 4 sessions.

For the 13 patients for whom brain mapping was performed for language, the same language eloquent areas were identified, regardless of the DO 80 presentation used (computer tablet, 2D VR, or 3D VR). However, for 1 patient (patient 13), the results were unclear in some areas for DO 80 on the computer tablet (hesitation or delay in denomination) that clearly were not eloquent according to assessment with the VRH. Eye tracking was functional, making it possible to trace the gaze of the patient during the task. During the DO 80 task, we noted that patients did not read the sentence “this is...,” instead saying it automatically.

Among the 10 patients who were able to perform the visuospatial and social VR experience without difficulty before surgery, 7 patients performed this task during brain mapping by DES and 2 patients (patients 11 and 14) during closure without DES (Table 1). For 1 patient (patient 9), it was not possible to present the task at the end of surgery because of hemostasis problems. Without DES, the mean time taken to identify eye contact was 2.3 seconds (range 2.0-2.5 seconds), and the mean time taken to recognize and verbalize the facial emotion was 3.2 seconds (range 2.6-4.4 seconds; total time for the task: 5.5 seconds, range 4.9-7.0 seconds). This total time is, in reality, shorter because the test is stopped manually once the patient’s response has been heard. For 2 patients (patients 2 and 15), DES of an area in the right hemisphere disturbed visual exploration and delayed avatar identification.

Despite the discomfort associated with the awake surgery procedure, none of the patients experienced vertigo or any vegetative signs of VR sickness. EEG modifications (afterdischarge or spike-and-wave) were observed in 27% (4/15) of the patients during the standard brain mapping procedure (without VRH). The same abnormalities persisted during brain mapping+VRH in 3 of these patients. IOSs occurred in 13% (2/15) of the patients. Epilepsy was the first sign for these patients and neither of these two patients displayed EEG modifications during the brain mapping procedure. The IOSs observed were short motor seizures, disappearing rapidly after cortical irrigation with iced saline. IOSs occurred during DES, before using the VRH for one patient and during the VR task for the other.

According to the questionnaire completed after surgery by the patient, the neurosurgeon, and the anesthetist, the use of the VRH was not an issue during surgery. During 1 operation, the neuropsychologist found it difficult to position the VRH. All participants agreed to continue studying this approach.

## Discussion

### Principal Findings

VR is a domain with growing applications in the field of neuroscience. This computer technology generates realistic images, sounds, and other sensations that simulate a user’s physical presence in a virtual or imaginary environment. A person using a VRH can look around the artificial world, *move* within it, and interact with virtual features or items. As such, VRH provides a unique opportunity to combine the naturalness of everyday interactions with the experimental controls required during brain mapping procedures, paving the way for new brain mapping procedures for complex cognitive functions.

The extension of our initial prospective trial confirmed that VRH with eye tracking and immersive virtual experiences was safe for patients undergoing awake craniotomy and brain mapping using DES. None of the patients experienced VR sickness and we observed no sympathetic nervous activity reported for this syndrome [15-20]. On the basis of our personal experience and published data, we are convinced that this good tolerance was because of patient preparation and training [21-23]. In total, in the 2 studies (45 patients), we observed afterdischarges in 17 (38%) patients and IOSs in 11 (24%) patients, rates within the range reported in previous studies: 71% for afterdischarges [24] and 3.4% to 31% for IOSs [25-29]. The IOS rate cannot be explained by the use of VRH, as most seizures occurred before its implementation. Thus, these seizures are more likely to be owing to our brain mapping procedure, which always began with positive motor stimulation to calibrate DES intensity. Consistent with our hypothesis, a recent review showed that patients with positive mapping (detection of the functional cortical area) are at a higher risk of IOSs [30]. Patients with preoperative seizures were also found to be more susceptible to intraoperative or postoperative seizures; 8 of the 11 patients with intraoperative IOSs in our 2 studies fell into this category. Another explanation would be the use of perioperative EEG: there is some controversy concerning the effect of brain activity monitoring on the occurrence of IOSs. Nossek et al [31] compared the occurrence of IOSs in the presence and absence of electrocorticogram (ECoG) use and found that the use of ECoG was associated with an increase in the occurrence of IOSs. The authors suggested that this was probably because the surgeon tends to increase stimulation more liberally when ECoG is used than when it is not.

All these data indicate that the use of a VRH during brain mapping in awake surgery does not specifically increase the rate of IOSs. Nevertheless, we recommend several precautions to prevent seizures during the use of a VRH for brain mapping procedures, including a well-trained team and, although there is no consensus regarding its usefulness, intraoperative monitoring of brain electrical activity.

This trial also demonstrated the feasibility of using eye tracking in patients undergoing awake craniotomy and brain mapping using DES. One of our apprehensions was the potential interference caused by devices in the operating room emitting infrared light, such as the neuronavigation system for tracking gaze. No such interference was observed and we were able to track eye movements on one of the screens of the neuronavigational system. Eye tracking revealed that the patients never read the sentence “this is...” associated with the image in the DO 80 VR task. This observation suggests that the patient said the sentence automatically, focusing only on the naming task. In the future, it would be interesting to develop a new VR task through a VRH with eye tracking for the specific exploration of reading. We recognize that eye-tracking sensors can be used in combination with regular computer screens or tablets. However, the eye tracker in the VRH, which combines features of both mobile and remote setups, prevents the risk of losing calibration and improves the success rate for measurements of eye positions and movements. Furthermore, the use of a VRH immerses the patient in the VR task, which is completely isolated from the surrounding operating room.

This trial also explored the possibilities and limitations of the visuospatial and social VR experiment developed with animated synthetic avatars. Avatars are perceived in a similar manner to real human beings and can be used to explore the complex processes of nonverbal language, empathy, and theory of mind [32]. Moreover, avatars enable researchers to manipulate, in a selective manner, variables that cannot be independently investigated in naturalistic situations, allowing precise control of not only the intensity, kinetics, and type of emotion but also of facial physiognomy, race, sex, and age as a function of the paradigm used. Tasks for use in awake surgery must take about 5 seconds, corresponding to the duration of DES. During this period, the maximum number of faces that can be analyzed in a visual field of 110° is limited, even if faces have the spatial advantage of capturing attention, reflecting their particular saliency, as well as their social value. Therefore, we decided to include 5 avatars, 1 in the center and the others in the 4 quadrants of the visualized field, to allow sufficient spatial exploration. The simulated social interaction involves the patients making visual contact with an avatar looking at them and describing the automatically triggered facial emotion of the avatar or their feelings about the desire for communication or social contact expressed by the avatar. Direct gazes between 2 people are known to constitute a significant, engaging social signal in all cultures. Decoding the movement of gaze plays an important role in predicting intentions and can be regarded as an important element in the theory of mind [33-36]. The patient’s eye-tracking data were used for real-time control of the virtual character’s emotional behavior in response to the participant’s gaze. We showed that this VR experience was compatible with the brain mapping procedure, with answers obtained within the maximum time allowable for DES. We observed that DES in some areas of the brain disturbed the answer to the test. Through eye tracking and patients’ answers, it was possible to attribute these failures to difficulties in exploring the space, identifying eye contact, or recognizing the facial emotion expressed or the associated mental state. A prospective study (ClinicalTrials.gov NCT04288505) is currently underway to determine the specificity and sensitivity of this new visuospatial and social VR task before its introduction into routine use for exploring the neural substrates of visuospatial and social functions during awake brain surgery. Several established pencil-and-paper tests are being used to determine its performance, including the bells test [37] for visuospatial attention functions and the Ekman test [38] and the *Reading the Mind in the Eyes* test [39] for social cognitive functions.

### Limitations

One of the difficulties in the field of VR research is the rapid progress of technology and the regular release of new VRHs. At the beginning of our research on the use of VR in the operating room, intending to detect hemianopsia and unilateral neglect during DES, we used the Oculus VRHs DK1 and DK2 (visual field 100°, resolution 1280×800 pixels, and refresh rate 60 Hz; Oculus) [4]. For the tolerance study, we chose to use a wireless low-cost, high-quality, customizable device: the Samsung Gear VR combined with a Samsung S7 smartphone (Android platform; visual field 96°, resolution 1440×1280 pixels, and refresh rate 60 Hz) [9]. However, before the completion of this study, new VRH models, including an eye-tracking system, became available. We chose to prolong the study with a higher-performance VRH, the HTC VIVE (larger visual field 110°, better resolution 2160×1200 pixels, and higher refresh rate 90 Hz) combined with an eye-tracking device (Tobii Pro SDK) capable of tracking the full HTC VIVE field of view and measuring the pupil.

### Conclusions

This study extended an initial prospective trial designed to confirm the feasibility and safety of VRH use and immersive virtual experiences for patients undergoing awake craniotomy and brain mapping using DES. Its added value lies in using a latest generation VRH, including eye tracking and a VR task designed to simultaneously test visuospatial and social functions.

## Appendix

Multimedia Appendix 1 Questionnaires completed by the patients and medical professionals.

## Abbreviations

DES: direct electrical stimulation
ECoG: electrocorticogram
EEG: electroencephalogram
fMRI: functional magnetic resonance imaging
IOS: intraoperative seizure
VR: virtual reality
VRH: virtual reality headset
