# Supplementary material for: Immersive Virtual Reality and Ocular Tracking for Brain Mapping During Awake Surgery: Prospective Evaluation Study
Source: J Med Internet Res. 2021 Mar 24;23(3):e24373. doi: 10.2196/24373 (PMC8074984; doi:10.2196/24373)
Supplement: Multimedia Appendix 1 [file jmir_v23i3e24373_app1.doc]

Clinical trial (NCT03010943)

Patient questionnaire

Date: /____/____/________/

As part of the study in which you agreed to participate, which concerns the evaluation of virtual reality headset use during awake surgery, we would like to record your impressions and any undesirable effects that you may have felt.

Please take a few minutes to complete this short questionnaire.

1. I find wearing the virtual reality headset unpleasant (Please tick the one that best applies):

1 2 3 4 5

○ ○ ○ ○ ○

Totally agree

Totally disagree

Tend to disagree

Neither agree nor disagree

Tend to agree

1. If asked, I would agree to perform awake surgery with a virtual reality headset again without hesitation (Please tick the one that best applies):

1 2 3 4 5

○ ○ ○ ○ ○

Totally agree

Totally disagree

Tend to disagree

Neither agree nor disagree

Tend to agree

1. Did you experience any side effects while wearing the virtual reality headset?

□ Yes □ No

1. If you answered “yes” to question 3, please check the option (s) best describing the side effects that you experienced:

□ Nausea

□ Vertigo

□ Discomfort

□ Anxiety

□ Pain

□ Seizures

□ Only with 3D images

□ Other, specify: …………………………………………………………………………………….

Clinical trial (NCT03010943)

Neuropsychologist questionnaire

Date: /____/____/________/

As part of the study concerning the evaluation of virtual reality headset use during awake surgery, we would like you to provide your impressions and indicate any difficulties you may have observed.

Please take a few minutes to complete this short questionnaire.

1. I think that the use of a virtual reality headset does not interfere with language assessment (Please tick the one that best applies):

1 2 3 4 5

○ ○ ○ ○ ○

Totally agree

Totally disagree

Tend to disagree

Neither agree nor disagree

Tend to agree

1. If asked, I would agree to perform awake surgery with a virtual reality headset again without hesitation (Please tick the one that best applies):

1 2 3 4 5

○ ○ ○ ○ ○

Totally agree

Totally disagree

Tend to disagree

Neither agree nor disagree

Tend to agree

1. Did you notice any side effects when the patient was wearing the virtual reality headset?

□ Yes □ No

1. If you answered “yes” to question 3, please tick the option (s) that best describe the side effects:

□ Nausea

□ Pain

□ Anxiety

□ Afterdischarges on EEG

□ Focal seizures

□ Generalized seizures

□ Only with 3D images

□ Other, specify: …………………………………………………………………………………….

Signed by: _______________________________________________

Clinical trial (NCT03010943)

Anesthetist questionnaire

Date: /____/____/________/

As part of the study concerning the evaluation of virtual reality headset use during awake surgery, we would like you to provide your impressions and indicate any difficulties you may have observed.

Please take a few minutes to complete this short questionnaire.

1. I think that the use of a reality headset results in an examination that does not interfere with anesthesia (Please tick the one that best applies):

1 2 3 4 5

○ ○ ○ ○ ○

Totally agree

Totally disagree

Tend to disagree

Neither agree nor disagree

Tend to agree

1. If asked, I would perform awake surgery with a virtual reality headset again without hesitation (Please tick the one that best applies):

1 2 3 4 5

○ ○ ○ ○ ○

Totally agree

Totally disagree

Tend to disagree

Neither agree nor disagree

Tend to agree

1. Did you notice any side effects when the patient was wearing the virtual reality headset?

□ Yes □ No

1. If you answered “yes” to question 3, please tick the option (s) that best describe the side effects:

□ Nausea

□ Pain

□ Anxiety

□ Afterdischarges on EEG

□ Focal seizures

□ Generalized seizures

□ Only with 3D images

□ Other, specify: …………………………………………………………………………………….

Signed by: _______________________________________________

Clinical trial (NCT03010943)

Neurosurgeon questionnaire

Date: /____/____/________/

As part of the study concerning the evaluation of virtual reality headset use during awake surgery, we would you to provide your impressions and indicate any difficulties you may have observed.

Please take a few minutes to complete this short questionnaire.

1. I think that the use of a virtual reality headset does not interfere with language assessment (Please tick the one that best applies):

1 2 3 4 5

○ ○ ○ ○ ○

Totally agree

Totally disagree

Tend to disagree

Neither agree nor disagree

Tend to agree

1. If asked, I would agree to perform awake surgery with a virtual reality headset again without hesitation (Please tick the one that best applies):

1 2 3 4 5

○ ○ ○ ○ ○

Totally agree

Totally disagree

Tend to disagree

Neither agree nor disagree

Tend to agree

1. Did you notice any side effects when the patient was wearing the virtual reality headset?

□ Yes □ No

1. If you answered “yes” to question 3, please tick the option (s) that best describe the side effects:

□ Nausea

□ Pain

□ Anxiety

□ Afterdischarges on EEG

□ Focal seizures

□ Generalized seizures

□ Only with 3D images

□ Other, specify: …………………………………………………………………………………….

Signed by: _______________________________________________
